# Internal Hernia Through a Mesoappendix Defect: A Case Report and Review of the Literature

**DOI:** 10.7759/cureus.79396

**Published:** 2025-02-21

**Authors:** Rafique Umer Harvitkar, Sugunesh Nanjan, Ioannis Hannadjas, Mariyam Shaheed, Alfredo Tonsi

**Affiliations:** 1 General Surgery, Royal Sussex County Hospital, Brighton, GBR; 2 Surgery, Royal Sussex County Hospital, Brighton, GBR; 3 Gastrointestinal Surgery, Royal Sussex County Hospital, Brighton, GBR

**Keywords:** closed loop obstruction, internal hernia, laparoscopy, mesoappendix, small bowel obstruction

## Abstract

Internal hernias are an uncommon cause of small bowel obstruction (SBO). If not identified and treated promptly, they can lead to severe complications. Among these, herniation through a mesoappendix defect is exceedingly rare. To our knowledge, SBO due to a mesoappendiceal defect has only been described in a limited number of cases. In this case, a 76-year-old woman presented to the emergency department with a two-day history of acute lower abdominal pain, accompanied by nausea, vomiting, and abdominal distension. Diagnostic imaging revealed a partial small bowel obstruction with two transition points. During diagnostic laparoscopy, a loop of proximal ileum was found herniating through a mesoappendix defect, resulting in a closed-loop obstruction. The herniated bowel was viable and was successfully reduced, followed by an appendicectomy. The postoperative recovery was uneventful. This case highlights the need to consider internal hernias in the differential diagnosis of SBO, even in patients with prior abdominal surgeries.

## Introduction

Internal hernias are a rare cause of small bowel obstruction (SBO), with an incidence of less than 1.5% in the general population. If untreated, they may result in a mortality rate of up to 48%. They account for approximately 0.6% to 6% of all SBO cases [1-4]. An internal hernia occurs when a viscus protrudes through a peritoneal or mesenteric aperture within the peritoneal cavity. These apertures can be congenital or acquired, with the latter often arising from surgical procedures, trauma, or inflammatory processes.

The mesoappendix, a peritoneal fold attaching the appendix to the ileum and caecum, is an uncommon site for internal herniation. Defects in the mesoappendix are rare and may be congenital or acquired [2]. When present, they can allow loops of the small intestine to herniate through, potentially causing bowel obstruction. To the best of our knowledge, only a few cases of SBO due to a mesoappendiceal defect have been reported. Given its rarity, this condition presents a diagnostic challenge and is often overlooked in the initial assessment of SBO.

Here, we report the case of a 76-year-old woman who presented with acute SBO secondary to an internal hernia through a mesoappendiceal defect. This case underscores the importance of considering internal hernias in patients presenting with bowel obstruction, even in those with a history of prior abdominal surgery.

## Case presentation

A 76-year-old woman presented to the emergency department with a two-day history of acute onset lower abdominal pain. The pain was constant, severe, and predominantly located in the lower abdomen. She also reported associated symptoms of nausea, vomiting, and progressive abdominal distension. No previous episodes of similar symptoms were noted. Her medical history was significant for a laparoscopic hysterectomy performed several years prior. She had no history of bowel surgery, abdominal trauma, or known hernias. She was not on any regular medications and had no known allergies.

On clinical evaluation, the patient appeared in distress due to pain. Her vital signs were as follows: blood pressure of 135/80 mmHg, heart rate of 95 beats per minute, respiratory rate of 18 breaths per minute, and a temperature of 37.2°C. Abdominal examination revealed a distended abdomen with generalized tenderness, more pronounced in the lower quadrants; bowel sounds were absent. There were no palpable masses or signs of peritonitis. Laboratory investigations showed a lactate level of 3 mmol/L, a white blood cell count of 16 × 10⁹/L, a C-reactive protein (CRP) level of 65 mg/L, and serum creatinine indicating stage 1 acute kidney injury (AKI). Given her surgical history, an adhesional SBO was initially suspected.

**Figure 1:**
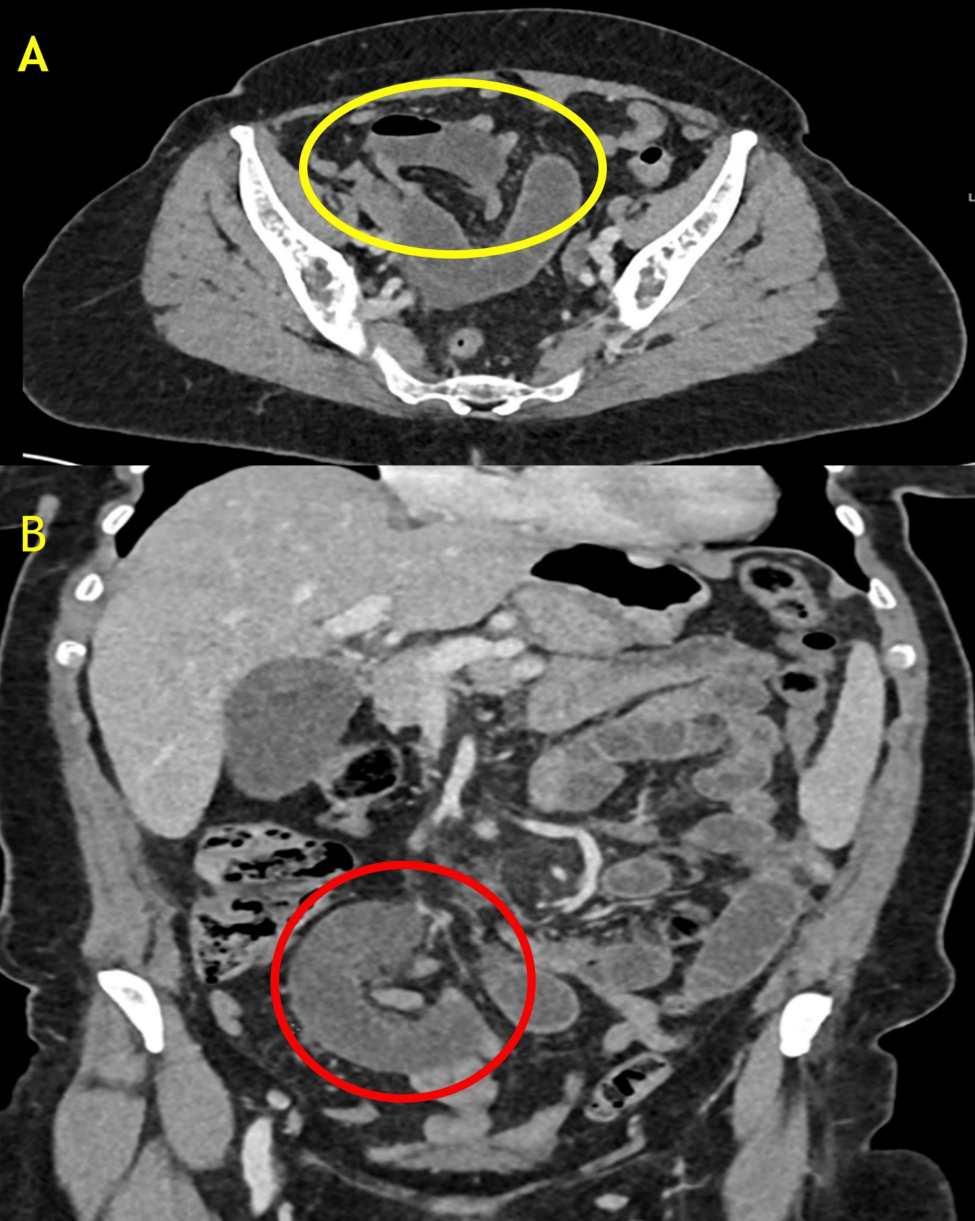
Contrast-enhanced CT images demonstrating small bowel closed-loop obstruction. (A) Axial section showing clustered, dilated small bowel loops with a characteristic "U-shaped" configuration, suggestive of a closed-loop obstruction (yellow circle). (B) Coronal section highlighting the transition point with mesenteric congestion and a radial distribution of mesenteric vessels converging towards the site of obstruction (red circle), consistent with internal herniation.

The patient underwent a contrast-enhanced computed tomography scan of the abdomen and pelvis (CTAP), which revealed a partial SBO with dilated loops measuring up to 26 mm (Figures 1A, 1B). There was evidence of pelvic ascites and mesenteric vascular engorgement. Notably, two transition points were identified: one in the midline pelvis and another to the left of the midline. Importantly, abnormal enhancement of the small bowel was preserved, suggesting of ischemia. Given the imaging findings and the patient’s clinical presentation, a decision was made to proceed with diagnostic laparoscopy.

**Figure 2:**
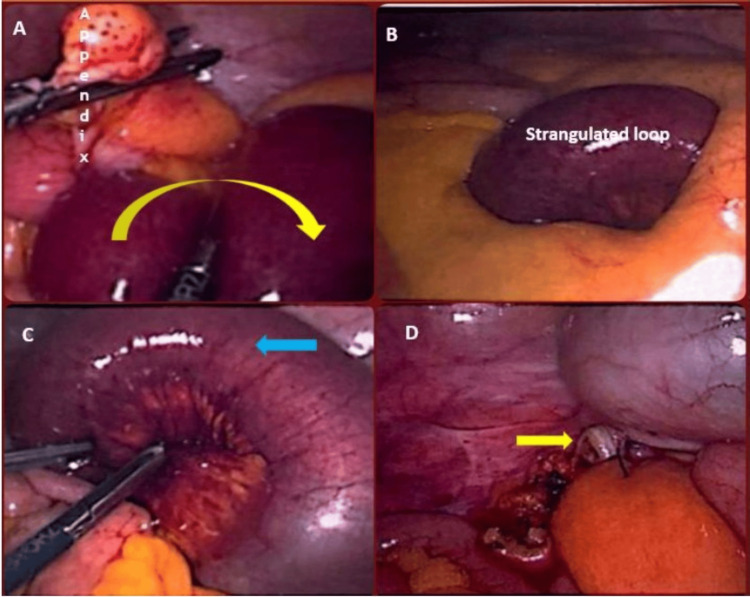
Intraoperative images: (A) an intraoperative view showing the appendix (labelled) with herniated small bowel loops entrapped along the mesoappendix (yellow curved arrow). (B) Strangulated small bowel loop visible after the initial reduction attempt, with signs of compromised vascularity. (C) Close-up of the reduced small bowel loop showing congestion and early ischemic changes (blue arrow) post-release of the obstruction. (D) Post-hernia reduction, the appendix is removed via laparoscopic appendicectomy (the yellow arrow indicates the appendiceal stump).

The patient was taken to the emergency operating theatre for a diagnostic laparoscopy within a few hours of admission. Intraoperatively, a loop of proximal ileum was found herniating through a defect in the mesoappendix, resulting in a closed-loop obstruction (Figures 2A, 2B). Although the herniated bowel initially appeared compromised in terms of blood supply, its appearance improved immediately upon reduction from the mesoappendix defect (Figure 2C). Therefore, bowel resection was not deemed necessary. Given the presence of a mildly inflamed appendix and to prevent future episodes, an appendicectomy was performed (Figure 2D). No other abnormalities or evidence of obstruction were noted during the procedure.

The patient tolerated the procedure well. Postoperatively, she was managed with intravenous fluids, analgesia, and prophylactic antibiotics. Her recovery was uneventful, with the return of normal bowel function and resolution of symptoms. She was discharged on the third postoperative day with advice for routine follow-up.

## Discussion

Internal hernias are a rare but potentially life-threatening cause of SBO. They result from the protrusion of the small intestine through a normal or abnormal peritoneal or mesenteric defect, leading to varying degrees of obstruction. The incidence of internal hernias is estimated to be less than 1% in the general population, though they account for 0.6% to 6% of all SBO cases. The most common types of internal hernias include paraduodenal (52%), pericaecal (12%), transmesenteric and transmesocolic (7%), foramen of Winslow (7%), intersigmoid (5.5%), and retroanastomotic hernias (4.5%). However, herniation through a mesoappendix defect is extremely rare and scarcely reported in the literature [1-6].

The mesoappendix is a peritoneal fold containing the appendicular artery and veins, connecting the appendix to the ileum and caecum. Defects in the mesoappendix can be congenital or acquired. Congenital defects arise due to incomplete embryological development, whereas acquired defects may result from prior inflammation, ischemia, or trauma. These defects may create a potential space through which a segment of the small intestine can herniate, leading to obstruction and, if untreated, strangulation and ischemia [1-3,7-9].

In this case, a loop of proximal ileum was found traversing through a mesoappendix defect, forming a closed-loop obstruction. Closed-loop obstructions are particularly concerning because they increase intraluminal pressure, impair venous drainage, and can rapidly progress to bowel ischemia. The presence of mesenteric engorgement and ascites on imaging further suggested vascular compromise.

Internal hernias often present with non-specific symptoms, making preoperative diagnosis challenging. Patients typically report colicky abdominal pain, nausea, vomiting, and distension, as seen in this case. The absence of previous bowel surgery may lower the suspicion for adhesional obstruction, making imaging crucial [6,10-12]. CT imaging remains the gold standard for diagnosing internal hernias, with sensitivity exceeding 82%. Key radiological findings to look for include dilated small bowel loops with transition points, whirling of mesenteric vessels, presence of ascites or mesenteric vascular engorgement, and abnormal clustering of small bowel loops [2,13].

A review of the literature highlights similar cases of herniation through a mesoappendix defect. In most cases, the herniated content was the terminal ileum, with patients predominantly presenting with small bowel obstruction (Table 1).

**Table 1:** Cases of herniation through a mesoappendix defect

S. no.	Year of publication	Age	Sex	Author(s)	Patient manifestation	Contents	Treatment given
1	1963	80	M	Rooney et al. [3]	Small bowel obstruction	Terminal Ileum	Reduction and closure
2	2016	5	M	Barman et al. [5]	Small bowel obstruction	Meckel's diverticulum	Small bowel resection
3	2020	55	M	Vinay et al. [7]	Small bowel obstruction	Terminal Ileum	Reduction and appendectomy
4	2021	33	F	Jones et al. [13]	Appendicitis	Ovary (right)	Reduction and appendectomy
5	2024	50	M	Gashey et al. [12]	Small bowel obstruction	Terminal Ileum	Reduction and appendectomy

The pathophysiology in these cases mirrors our findings, with congenital or acquired defects serving as the herniation site. Management consistently involves surgical intervention, with laparoscopy preferred due to its minimally invasive nature and diagnostic capabilities.

## Conclusions

This case underscores the importance of considering internal hernias in the differential diagnosis of SBO, even in patients with prior abdominal surgeries. Internal hernias through a mesoappendix defect represent a rare but serious condition that requires prompt recognition and surgical management. CT and diagnostic laparoscopy are crucial to prevent complications such as bowel ischemia and necrosis, hence improving patient outcomes.
